# Comparison and Cost Analysis of Drinking Water Quality Monitoring Requirements *versus* Practice in Seven Developing Countries

**DOI:** 10.3390/ijerph110707333

**Published:** 2014-07-18

**Authors:** Jonny Crocker, Jamie Bartram

**Affiliations:** The Water Institute, Gillings School of Global Public Health, University of North Carolina at Chapel Hill, 148 Rosenau Hall, CB #7431 Chapel Hill, NC 27599, USA; E-Mail: jonny.crocker@unc.edu

**Keywords:** drinking water, *E. coli*, coliform, monitoring, cost analysis

## Abstract

Drinking water quality monitoring programs aim to support provision of safe drinking water by informing water quality management. Little evidence or guidance exists on best monitoring practices for low resource settings. Lack of financial, human, and technological resources reduce a country’s ability to monitor water supply. Monitoring activities were characterized in Cambodia, Colombia, India (three states), Jordan, Peru, South Africa, and Uganda according to water sector responsibilities, monitoring approaches, and marginal cost. The seven study countries were selected to represent a range of low resource settings. The focus was on monitoring of microbiological parameters, such as *E. coli*, coliforms, and H_2_S-producing microorganisms. Data collection involved qualitative and quantitative methods. Across seven study countries, few distinct approaches to monitoring were observed, and in all but one country all monitoring relied on fixed laboratories for sample analysis. Compliance with monitoring requirements was highest for operational monitoring of large water supplies in urban areas. Sample transport and labor for sample collection and analysis together constitute approximately 75% of marginal costs, which exclude capital costs. There is potential for substantive optimization of monitoring programs by considering field-based testing and by fundamentally reconsidering monitoring approaches for non-piped supplies. This is the first study to look quantitatively at water quality monitoring practices in multiple developing countries.

## 1. Introduction

Access to safe drinking water can prevent disease outbreaks, and lower diarrheal and other disease burden. Approximately 768 million people across the world lack access to an “improved” drinking water source [1], though an even larger number use a water supply that is unsafe or has an elevated sanitary risk (3.06 billion) [2]. Delivering drinking water free of pathogens depends on hazard control, treatment, safe distribution, and monitoring. While drinking water quality management has an extensive history, the first formal frameworks for ensuring safe drinking water were developed based on the Hazard Analysis and Critical Control Point (HACCP) system for food safety assurance [3]. The World Health Organization (WHO) adapted the HACCP framework specifically for drinking water safety [4]. One component of the WHO framework for safe drinking water is a “Water Safety Plan” (WSP), which comprises system assessment, operational monitoring, and management planning [5].

The WHO Guidelines for Drinking-water Quality [4] and WSP manual [5] recommend that both operational monitoring and independent surveillance should take place; both normally include testing for indicators of fecal contamination. Operational monitoring serves to inform decision making and corrective actions regarding control measures (e.g., source protection, water treatment), while surveillance of drinking water quality engages an independent third party in oversight of water supply, with the specific mandate for protection of public health. This study focused on operational monitoring and surveillance (here collectively referred to as “monitoring”), which are necessary components of water quality management.

Little published literature exists on evidence for the efficiency or effectiveness of monitoring as practiced. Case studies in Uganda and Peru [6] investigated how to conduct effective drinking water surveillance in urban settings, and recommended that surveillance should be used primarily for protection of public health, and also to target water supply improvements to where they are most needed. A more recent study [7] mapped the institutions involved in monitoring from nine developing countries, of which Cambodia and Peru are included in this study. They concluded that urban water supply is better resourced and more frequently monitored than informal settlements and rural supplies. They found that surveillance monitoring sometimes involves auditing water supplier test results rather than direct testing of water supplies.

To our knowledge, there are no studies that examine compliance with drinking water quality monitoring requirements, or that analyze the costs of monitoring programs in low and middle income countries. This paper addresses this gap by characterizing monitoring requirements and practices, estimating marginal costs, and identifying financial constraints across seven countries selected to reflect a range of water supply conditions. Study countries are Cambodia, Colombia, India (three states), Jordan, Peru, South Africa, and Uganda.

## 2. Methods

### 2.1. Country Selection

Selected countries form a stratified sample across water and sanitation country clusters [8], which were designed for country selection in multi-country studies to represent a range of water supply and sanitation conditions. Study countries were selected from clusters 2–5, which together include 118 developing and middle-income countries. Cluster 1 was intentionally excluded as it is comprised of 33 developed and industrialized countries, which were not the focus of this study. The selected countries come from all major geographic regions. As government functions for drinking water in India are decentralized to the state level, three of the most populous states in India were included in the study: Maharashtra, Uttar Pradesh, and West Bengal. These three states also represent a range of the total proportion of the population that lives in rural areas, which relates to the proportion of drinking water accessed from non-piped supplies; and of Gross Domestic Products (GDP), which is a measure of economic activity and captures the value of goods and services.

### 2.2. Data Collection

Gray literature consisting of water policies, regulations, standards, guidelines, and manuals was collected from the internet and by correspondence with stakeholders involved in water quality management in each study country. Field work was conducted in 2010 by a team of five researchers. Field work involved focus groups, interviews, and surveys with employees of organizations involved in monitoring; observation of laboratories and sampling trips; and collection of laboratory testing records.

Focus group interview guides were piloted in March 2010 by four of five researchers. Higher-level employees such as directors were interviewed for broad information such as organizational responsibilities. Lower level employees such as laboratory technicians were asked for details such as samples collected per day and sample analysis methods. Observation of sampling and sample analysis allowed for estimation of time spent on each task. Multiple interviews and observations from within each organization were used to verify data.

A minimum of six interviews and two observations of monitoring being practiced were completed per study site. Details on data collection activities for each study site are shown in Table 1. All data collection methods were submitted to the University of North Carolina Office Institutional Review Board (IRB) for approval before data collection began. This study was determined to not constitute “human subjects research” and received an exemption from the IRB.

### 2.3. Characterization of Monitoring Approaches

Organizations were mapped based on their roles, responsibilities, and interactions in water quality monitoring following the model developed in a 2011 study [7]. Monitoring practices were grouped into separate “scenarios”, differentiated by monitoring purpose, sample collection and transport method, and analytical method. Institutional maps, scenario descriptions, and scenario parameters for each country can be found in Supplement 1.

**Table 1:** Data collection activities.

Site	Interviews	Observations *
Cambodia	20	3
Colombia	6	2
India—Maharashtra	11	4
India—Uttar Pradesh	11	4
India—West Bengal	6	3
Jordan	13	3
Peru	14	2
South Africa	20	4
Uganda	13	2

* Observations include laboratories, treatment plants, and sample collection trips.

Monitoring requirements—“prescribed” monitoring—were identified by reviewing government policies, regulations, and standards. Prescribed monitoring was population-based in every country. Population-based monitoring frequencies were multiplied by population and water supply coverage data to arrive at the total number of tests prescribed annually for each monitoring scenario.

Monitoring as practiced—here forth called “extrapolated monitoring”—was calculated from a review of monitoring reports, interviews, and observations. Monitoring reports contain the number of samples analyzed for a given water supply, laboratory, or geographic area for a fixed period of time (often monthly, quarterly, or annually). Interviewees could often report figures that allowed for calculating testing figures, for example, the average number of samples collected per day for a given laboratory. Additionally, observations such as watching a day of sampling or laboratory analysis revealed how many samples could be collected and analyzed in a day. For most scenarios, data were collected for one region of a country, or for one laboratory. Country-wide, annual monitoring figures were estimated by extrapolation. For example, for rural surveillance monitoring in Peru, the number of tests per capita for the La Libertad region was obtained through interviews then extrapolated to all of Peru using the rural population of the whole country. The parameters used to calculate prescribed monitoring figures, and to estimate monitoring practice are listed in Table 2.

**Table 2:** Parameters used to calculate monitoring as prescribed and estimate monitoring as practiced.

Prescribed Testing *	Extrapolated Testing *
Monitoring standards	Tests/laboratory
Settlements by size	Tests/technician
Water supplies by size	Tests/region
	Compliance with standards
	Laboratories
	Settlements by size
	Water supplies by size
	Population census

* Data sources used to calculate prescribed testing and extrapolated monitoring as practiced are listed by country in Supplement 2.

Extrapolated testing practice figures were triangulated by comparing figures reported from different interviews, numbers of tests in water authority reports, and testing records from laboratory notebooks.

### 2.4. Cost Analysis

Marginal costs include sample collection and transport; materials for sample testing; and labor associated with sample collection, transportation, and sample analysis. Capital and capital maintenance costs were excluded, as they were not considered costs that would increase with increased compliance with testing requirements. Parameters used to calculate costs are listed in Table 3.

**Table 3:** Parameters used to calculate costs for each monitoring scenario.

Test Materials	Labor	Transportation
Test cost *	Staffing	Vehicles type and number
	Tests/technician	Hours/day sampling
	Samples/sampler	Distances traveled
	Salaries *	Sampler reimbursements

* When country-specific costs were not captured during field work, costs cited in literature were used. The costs used for each parameter by country are listed in Supplement 2.

Costs for each category were calculated differently depending on the data available for each scenario. For example, for some scenarios water utilities were able to provide monthly sample transportation budgets, while in other cases the distance traveled and cost per mile were estimated based on the amount of time spent sampling each day, and the vehicles used for sampling. The data used for each country are described in Supplement 2.

When a specific price for test materials was not obtained in a country, values from the literature were used. Kromoredjo and Fujioka [9] evaluated three most probable number (MPN) test methods; Colilert, Laurel-Tryptose Broth + 4-methylumbelliferyl-β-d-glucuronide (LTB + MUG), and H_2_S test strips. The costs for these tests were reported as $6.50, $1.62, and $0.62 respectively. A study from the Massachusetts Institute of Technology reported membrane filtration test materials at unit price of $2.25, and Petrifilm test materials at $1.04 [10]. Bain *et al.* [11] completed a catalogue of all common microbial drinking water tests, and found that material costs varied from $0.60 to $5.00 for presence absence tests, and from $0.50 to $7.50 for quantitative tests. Test materials costs are presented in Table 4. The $0.62 figure for H_2_S presence-absence, $1.62 for MPN, and $2.25 for membrane filtration are used in this study. These figures are within the ranges reported by Bain *et al.* [11].

**Table 4:** Test materials cost figures from published literature.

Test Type	Indicator	Materials Cost	Source
H_2_S test strips	Presence-absence	$0.62	[9]
Multiple tube	MPN	$1.62	[9]
Colilert kit	MPN	$6.50	[9]
Petrifilm	Colony forming units (CFU) per 1 mL	$1.04	[10]
Membrane filtration	CFU per 100 mL	$2.25	[10]
Various	Presence-absence	$0.60–$5.00	[11]
Various	MPN or CFU	$0.50–$7.50	[11]

The cost for an individual test within each country and scenario was multiplied by the number of prescribed or extrapolated tests in each scenario to arrive at the total cost of a scenario. Capital costs such as laboratory construction, as well as laboratory maintenance, labor not associated directly with microbial monitoring, and legislation and standards development were not included in cost analysis. These excluded costs were assumed to exist regardless of the extent of prescribed or actual monitoring for microbial parameters.

## 3. Results

Each of the three states from India is weighted as an individual country, thus the term “study sites” here refers to study countries plus Indian states. Results are averaged across study sites. Results are site-averaged rather than population-averaged, otherwise the Indian states would dominate the results because of their high populations. Characteristics of the study sites are presented in Table 5. GDP is presented in United States dollars in 2010 (2010$).

**Table 5:** Descriptive statistics for study sites.

Country	Population	Access to an Improved Drinking Water Source (%)	Population Living in a Rural Setting (%)	GDP Per Capita (2010$)
India **	1,156,897,766	88	70.5	976
Uttar Pradesh *	166,198,000	81	79.2	323
West Bengal *	80,176,000	73	72.0	618
Maharashtra *	96,879,000	72	57.6	905
Jordan **	6,113,000	98	17.0	2654
Colombia **	46,043,696	92	24.2	3648
Peru **	29,797,694	82	25.4	3880
Uganda **	32,369,558	67	85.2	403
Cambodia **	14,521,275	61	79.0	598
South Africa **	49,052,489	91	43.0	5826

* [12], ** [13].

### 3.1. Monitoring Scenarios

For each study site, monitoring requirements in policies, regulations, and standards are set by the government. For those sites that require both operational monitoring and surveillance, operational monitoring involves more frequent sampling, more sampling points (source water through to point of use), and uses a wider range of tests and laboratories than does surveillance. For operational monitoring, the largest cities have dedicated laboratories (which are used only for drinking water monitoring) and collect and process many samples. Small cities and towns use shared laboratories (which are also used for purposes besides drinking water monitoring), and collect fewer samples and at lower frequencies. Operational monitoring of rural sites with point-source water supplies follows the same approach as for small cities, if they are operationally monitored at all. Surveillance monitoring is generally run by health departments, and relies on decentralized laboratory networks to monitor all water supplies.

Monitoring approaches were reportedly based on a few guidelines—such as those from the International Standards Organization, the US Environmental Protection Agency, and the WHO. Interviewees indicated that standards are infrequently reviewed, and rarely changed when they are reviewed, thus rarely reflect technological advances in testing. For example, in Peru the water quality policy and regulation had been in place since 1946, and while likely still useful, there have been advances in testing technologies and updates in international guidelines since then.

**Figure 1:**
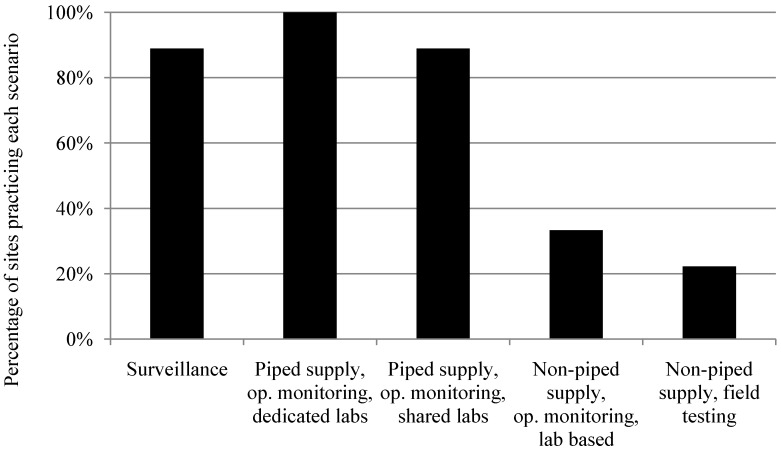
Scenario prevalence across nine study sites.

Operational monitoring of piped water supplies using dedicated laboratories is the most common scenario, practiced in all nine study sites. The next most common scenarios are operational monitoring of piped water supplies using shared laboratories and surveillance, which occur in eight of nine study sites. Operational monitoring of non-piped, or point-source water supplies (such as boreholes, which are generally rural) is rare. Where operational monitoring of non-piped water supplies is not prescribed, these supplies are either only monitored as part of surveillance, or not monitored at all. Overall scenario prevalence across the nine study sites (Figure 1) is here forth categorized into operational monitoring of large water supplies, operational monitoring of small water supplies, and surveillance monitoring of all water supplies within a country for further analysis. The cutoff between large and small supplies varies slightly by country, depending on locally adopted definitions and data availability. Descriptions of what is included in each of the scenarios can be found in Supplement 1.

### 3.2. Prescribed versus Extrapolated Monitoring Practice

Drinking water quality monitoring requirements vary between scenarios. The average number of tests required per year per capita is highest for surveillance monitoring (12.09 tests/year/1000 capita), followed by operational monitoring of small supplies (8.28 tests/year/1000 capita), and lowest for operational monitoring of large supplies (2.53 tests/year/1000 capita served). The required number of tests per capita is highest for surveillance because, though operational monitoring testing frequencies are higher, non-piped water supplies are not commonly operationally monitored but are covered by surveillance in eight of nine study sites—South Africa being the exception.

**Figure 2:**
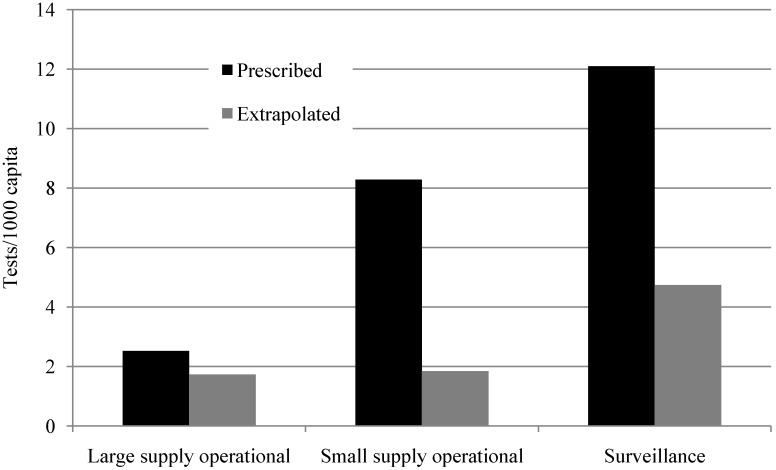
Prescribed and extrapolated levels of monitoring by scenario.

Monitoring in practice differs from the monitoring as prescribed by the government in the number of samples collected and analyzed. Figure 2 shows prescribed *versus* extrapolated actual practice for monitoring across the three main scenarios for all nine study sites. The extrapolated actual number of tests completed each year per capita is less than prescribed for all three scenarios. Compliance with monitoring requirements is the highest for operational monitoring of large supplies (77.2%), followed by operational monitoring of small water supplies (59.7%), with surveillance monitoring having the lowest site-averaged compliance (53.3%). Though the testing frequency and number of tests *per water supply* is the highest for operational monitoring of large supplies, the number of tests per capita is the lowest.

**Figure 3:**
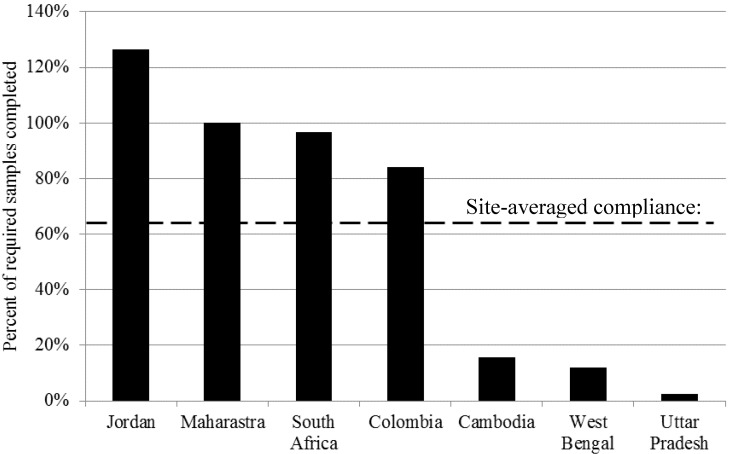
Compliance with monitoring requirements by country.

Compliance with monitoring requirements varies across the seven study sites for which extrapolated practice results are available, shown in Figure 3. The site-averaged compliance is 63% of required tests completed annually. Four of seven sites have compliance over 80%, while three fall below 20%. Peru and Uganda are missing some data for extrapolated monitoring practice, so extrapolated monitoring practice and compliance figures exclude these two countries. The compliance figure for Cambodia includes only operational monitoring, as data for surveillance estimates were unavailable.

### 3.3. Cost Analysis

**Figure 4:**
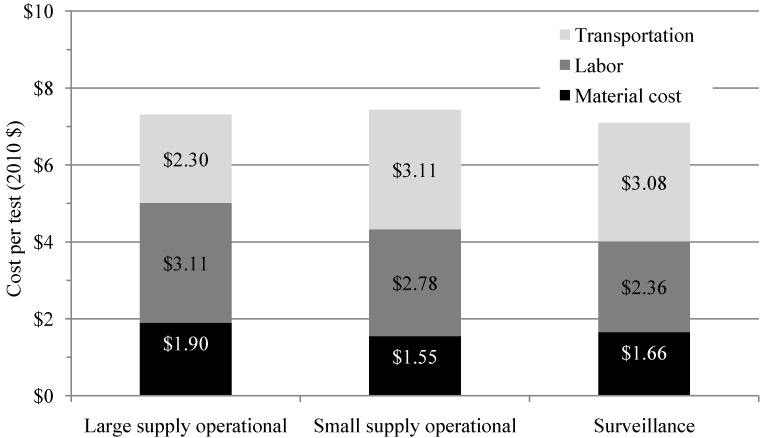
Marginal cost per test for three scenarios averaged across nine sites.

The marginal cost per test is similar across scenarios, ranging from $7.09 to $7.44. Monitoring of large supplies in urban settings is often associated with higher laboratory staff salaries, and employs sample analysis methods with more expensive material costs. Operational monitoring of small supplies and surveillance monitoring (often dominated by rural supplies) have significantly higher transportation costs. Cost breakdown by scenario is displayed in Figure 4.

Table 6 shows the prescribed and extrapolated site-averaged, minimum, and maximum levels of testing, costs, and compliance by scenario across the nine study sites. Testing requirements vary significantly across sites. For example, for operational monitoring of small supplies, 28.4 tests per 1000 capita are required per year in Uttar Pradesh where all non-piped supplies are to be tested twice a year, and only 0.61 tests per 1000 capita are required per year in Cambodia, where small piped supplies are to be tested 4 to 24 times per year depending on whether they are public or private, and non-piped supplies are not operationally monitored.

Water supplies that serve small populations require a higher number of tests per capita in all study countries. Compliance for small supply operational monitoring is 59.7%, and for surveillance compliance is 53.3%. These small supplies and point-sources are often spread far apart, making sample collection and transport costly. Monitoring at 100% compliance would cost an average of 130% more per capita for small supply operators compared to large supply operators, and an average of 730% more per capita for surveillance agencies compared to large supply operators.

**Table 6:** Prescribed and extrapolated levels of testing and testing costs by scenario averaged across nine study sites.

Parameter		Large Supply Operational	Small Supply Operational	Surveillance
Prescribed	# sites *	*9 sites*	*9 sites*	*7 sites*
	Cost/test (2010$)	7.31 [5.43, 10.4] **	7.44 [1.10, 10.11]	7.09 [4.79, 9.14]
	Tests/1,000 capita	2.53 [1.20, 6.34]	8.28 [0.61, 28.4]	12.1 [1.68, 33.4]
	Cost/1000 capita (2010$)	18.0 [6.52, 39.3]	45.4 [5.87, 204]	84.6 [8.03, 270]
Extrapolated	# sites *	*7 sites*	*7 sites*	*6 sites*
	Compliance (%) ***	77.2 [5.31, 167]	59.7 [0.27, 170]	53.3 [11.0, 100]
	Tests/1000 capita	1.73 [0.064, 5.42]	1.84 [0.078, 5.38]	4.74 [0.68, 21.5]
	Cost/1000 capita (2010$)	12.9 [0.41, 40.7]	15.6 [0.081, 49.2]	29.2 [4.02, 125]

* “Sites” refers to countries and Indian states; ** Bracketed values are minimums and maximums; *** Compliance refers to extrapolated number of samples analyzed compared to number of test required.

## 4. Discussion

### 4.1. Limited Monitoring Scenarios Practiced

Monitoring approaches encountered tend to be based on a few guidelines which may not be tailored to the range of settings and types of water supply seen in the countries studied. The most frequently seen monitoring scenarios across study sites were operational monitoring for piped water supplies using dedicated or shared laboratories, followed by surveillance of all water supplies, and lastly operational monitoring for non-piped supplies. There are a variety of reasons why this may be the case. Piped supplies serve large populations, thus are a more cost-effective use of limited resources, as drinking water for populations into the millions can be monitored by testing one water supply. Piped supplies are better equipped—with treatment plants or operators—to respond to monitoring data which identifies contamination. Lastly, collecting samples is easier for piped supplies than for non-piped supplies, as they are often closer to laboratories or have on-site laboratories, or are in close proximity to other water supplies. The implication is that the non-piped supplies which are more likely to be contaminated [14] receive the least monitoring attention.

### 4.2. Gap between Prescribed and Extrapolated Monitoring

The biggest gap between prescribed monitoring and extrapolated actual practice is for monitoring of low population density areas (small supply operational and surveillance scenarios), where compliance is 59.7% and 53.3% respectively. The gap between prescribed and extrapolated monitoring practice may be due to the variation in cost per capita by scenario type caused by testing requirements and distance between sampling points. Though the testing frequency and number of tests *per water supply* required in national standards are the highest for operational monitoring of large supplies, the number of tests *per capita* is the lowest, as shown in Figure 2 and Table 6. This is because in large urban settlements, one water supply may serve millions of people, while small water supplies may serve as few as 1000 people but still require at least yearly testing. From Table 4, the three sites with the lowest compliance have the lowest GDP per capita and highest percentage of population living in rural settlements. It is possible that the cost of monitoring and the distances between sampling sites are major barriers to monitoring in these sites.

A study in 2011 [7] found that compliance with monitoring requirements is less common for smaller water supplies, though they do not quantify compliance in their study. Water supplies that serve smaller populations are more often unimproved [1], and while unimproved sources may sometimes provide safe water, they have much higher variation in quality [14] thus monitoring them is more likely to reveal contamination requiring action or prioritization of resources.

### 4.3. Costs of Monitoring

The marginal cost per test is very similar across the three scenarios categories. Test material costs are higher for operational monitoring of large supplies as the more expensive membrane filtration method is generally used, but expenditure on sample transport is lower due to sampling sites being closer to one another and to laboratories. Labor is more expensive in large urban areas, as staff often have lower salaries in rural areas. In the case of Uttar Pradesh in India, rural labor costs are further reduced by having volunteers within communities analyze water samples using field kits.

Transport and labor together are a major portion of the overall cost of monitoring. Compliance with monitoring requirements for all drinking water supply would be possible in low resource settings if labor and transportation costs associated with monitoring were reduced. The community-based rural monitoring scenario in Uttar Pradesh illustrates this, as volunteer teams test their own water sources, removing labor and transportation costs; though this introduces new challenges evident in the low compliance figures for small supplies in Uttar Pradesh, such as training and motivating volunteers and retrieving test results.

Field-based tests can be of similar accuracy to laboratory-based tests [15], and some have regulatory approval. Some portable tests are based on detecting H_2_S-producing microorganisms, which one study found can provide comparable quality to more traditional indicators such as coliform or *E. coli* given appropriate sample volumes [16]*.* Although no H_2_S tests have regulatory approval from the US EPA or International Organization for Standardization [14], some of the approved coliform and *E. coli* tests can be used in the field as presence-absence, or using portable incubators [17]. Incorporation of field-based testing into operational monitoring or surveillance could reduce the burden on organizations and agencies currently tasked with monitoring the small supplies and point sources.

A 2005 study in Europe estimated that 40 to 50 million people access drinking water from small or very small systems or private wells [18]. The US EPA estimated that in 2008 approximately 53 million people in the US accessed drinking water from small community water systems, which include private wells [19]. Hunter *et al.* modeled the costs and benefits of improving rural water supplies in developed countries [20], and found that there are sufficient health benefits to justify investment in improving rural supplies. Though the findings and conclusions presented here only pertain directly to developing countries, similar challenges to monitoring exist in developed countries, particularly where non-piped water supplies are common.

### 4.4. Study Limitations

Study sites were selected to represent the range of monitoring practices present in developing countries; no data were collected from developed or industrial countries. This study focuses on regularly occurring monitoring scenarios, and excludes other forms of monitoring such as one-time testing when new water supplies are developed.

Actual practice will vary from the results presented. Extrapolated practice is calculated from data collected from interviews, databases, and other means. Additionally, data are of limited geographic representation. This method of extrapolation may overestimate compliance, as laboratories and utilities with higher compliance are more likely to respond to interview requests. Material costs are not country-specific in most cases, as they are taken from literature, though a review of costs reported in the literature suggests little variation [9,10,11].

Cost analysis excludes capital costs (such as laboratory construction) and capital maintenance (such as repairs on laboratory equipment), though these costs may exist regardless of testing drinking water for microbiological parameters. For sites with the lowest compliance, existing laboratory infrastructure might not be sufficient to handle the number of tests required for 100% compliance. In these cases, attempting to reach 100% compliance would be infeasible without increasing laboratory capacity, changing the approach to monitoring, or changing the monitoring requirements themselves. Lastly, material costs from the literature do not account for the cost of customs duties and transportation to laboratories after purchase. These limitations in the cost-analysis may result in underestimating the costs of monitoring, which serves to strengthen the conclusions and recommendations of this study.

## 5. Conclusions

Drinking water quality monitoring as prescribed in national and state regulations and standards poses challenges to the organizations responsible for sample collection and analysis, and the shortfall is primarily in operational monitoring of small supplies and surveillance monitoring where risk is greatest. Compliance with monitoring requirements varies substantially between countries, from 3% to over 100% (Figure 3). In all study countries, there are few approaches to monitoring drinking water supply, and monitoring is driven by regulation. Current regulations for monitoring piped water supplies require expensive test methods that are normally conducted in static laboratories. A fundamental rethinking of monitoring approaches used for non-piped water supplies that serve low density populations could yield substantial savings, and increased benefits of monitoring. In many countries, yearly monitoring of every rural water supply is simply not possible, and would not enable prevention of contamination events. In such cases, less frequent monitoring or monitoring a sample of supplies might better serve decision makers. Monitoring approaches and associated sampling strategies for these water supplies should be revised to reflect what information can actually be used to ensure safe water delivery.

## Supplementary Files

Supplementary File 1 Supplementary Information (PDF, 1209 KB)
