# Supplementary material for: Comparison and Cost Analysis of Drinking Water Quality Monitoring Requirements versus Practice in Seven Developing Countries
Source: Int J Environ Res Public Health. 2014 Jul 18;11(7):7333–46. doi: 10.3390/ijerph110707333 (PMC4113879; doi:10.3390/ijerph110707333)
Supplement: Supplementary File 1 — Supplementary Information (PDF, 1209 KB) [file ijerph-11-07333-s001.pdf]

Supplementary Information

Comparison and Cost Analysis of Drinking Water Quality Monitoring Requirements versus Practice in Seven Developing Countries

---

Supplement 1—Individual Country Results

**1.**	**Cambodia**	**2**
**2.**	**Colombia**	**3**
**3.**	**India—National Level**	**4**
**4.**	**India—Maharasthra**	**5**
**5.**	**India—Uttar Pradesh**	**7**
**6.**	**India—West Bengal**	**8**
**7.**	**Jordan**	**10**
**8.**	**Peru**	**11**
**9.**	**South Africa**	**12**
**10.**	**Uganda**	**14**

1. Cambodia

1.1. Sector Description

Urban water supply involves the Ministry of Industry, Mines and Energy (MIME), which oversees two public autonomous agencies (the Phnom Penh Water Supply and the Siem Reap water authority), thirteen public water supply authorities, and approximately 100 licensed domestic private service providers (DPSPs). The Department of Potable Water Supply within MIME is responsible for the development of public water suppliers and regulates domestic private service providers through a licensing regime. The Department of Potable Water supply within MIME takes an audit-based approach to monitoring the two public autonomous agencies and the other public water suppliers; similarly it receives water quality analysis results directly from the DPSPs on a regular basis.

Rural water supply is overseen by the Ministry of Rural Development (MRD). International organizations and NGO's are supposed to sign a Memorandum of Understanding with the MRD to install water supplies. The MRD partners with organizations such as UNICEF to install rural water supplies. Although sampling frequencies and parameters are suggested in the Cambodian Drinking Water Quality Standards, they are generally not followed by the MRD for logistical reasons. Roles and responsibilities in the water supply sector in Cambodia are displayed in Figure S1.

Figure S1. Cambodia institutional map.

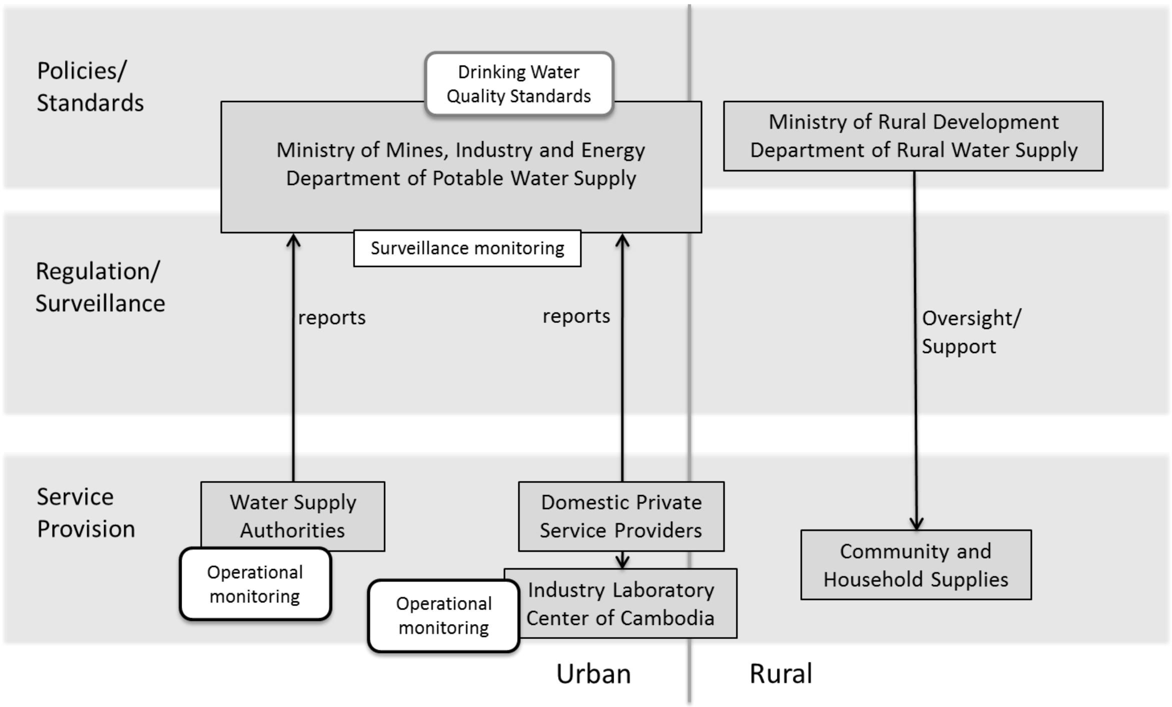

1.2. Scenarios

Large supply operational monitoring in the case of Cambodia includes Phnom Penh. The small supply operational monitoring scenario includes the 13 public utilities and approximately 100 DPSPs. Surveillance monitoring is in the mandate for the MRD, but no evidence was found on the number of

supplies supposed to be monitored, or the level of monitoring conducted by MRD. Quantitative values for monitoring scenarios in Cambodia are presented in Table S1.

Table S1. Cambodia scenarios parameters.

**Monitoring Type**	**Parameter**	**Large Supply Operational**	**Small Supply Operational**	**Surveillance**
	target pop	2,009,264	966,030	14,700,000
prescribed	tests/1000 capita	2.28	0.61	missing data
	cost/1000 capita	$23.66	$5.87	
extrapolated	tests/1000 capita	0.26	0.31	
	cost/1000 capita	$2.69	$2.93	

2. Colombia

2.1. Sector Description

The Ministry of Social Protection is the main organization in Colombia responsible for creating and amending drinking water legislation. They work with the Ministry of the Environment and the National Institute of Health (INS—Instituto Nacional de Salud) to perform this function. The National Institute of Health acts as the technical arm of the Ministry of Social Protection. They perform surveillance monitoring for all drinking water in Colombia. They operate a central reference laboratory, and oversee a network of laboratories across Colombia (public, private, and NGO) that support both surveillance and operational monitoring. They also act as the lead member of the committee that is responsible for determining drinking water quality monitoring protocol (*i.e.*, regulations and standards). The Superintendent of Public Utilities (SSPD—Superintendencia de Servicios Públicos Domiciliarios) is responsible for regulation of water suppliers, including regulation of water quality monitoring. Public and private suppliers (EPS—Empresa Prestadora de Servicios) in Colombia are required to monitor their drinking water at a frequency listed in Resolution #2115. These suppliers either operate their own laboratory, or send samples to one of the laboratories listed under the INS laboratory network. Results are used for operational purposes, and are additionally sent to the SSPD for compliance, and to the Ministries of Social Protection and of the Environment upon request.

Rural water supply that is not managed by an EPS are considered community supplies, and are monitored as part of surveillance by the INS. The INS, acting as the technical arm of the Ministry of Social Protection, is responsible for conducting surveillance monitoring for all water supply in Colombia. The INS draws upon a network of laboratories across the country which includes at least one INS laboratory per state as well as other public and private laboratories. These results are sent to the INS department of environmental health in Bogota. The frequency of sampling for surveillance monitoring is lower than that of operational monitoring, and is listed in Resolution #2115. Roles and responsibilities in the water supply sector in Colombia are displayed in Figure S2.

Figure S2. Colombia institutional map.

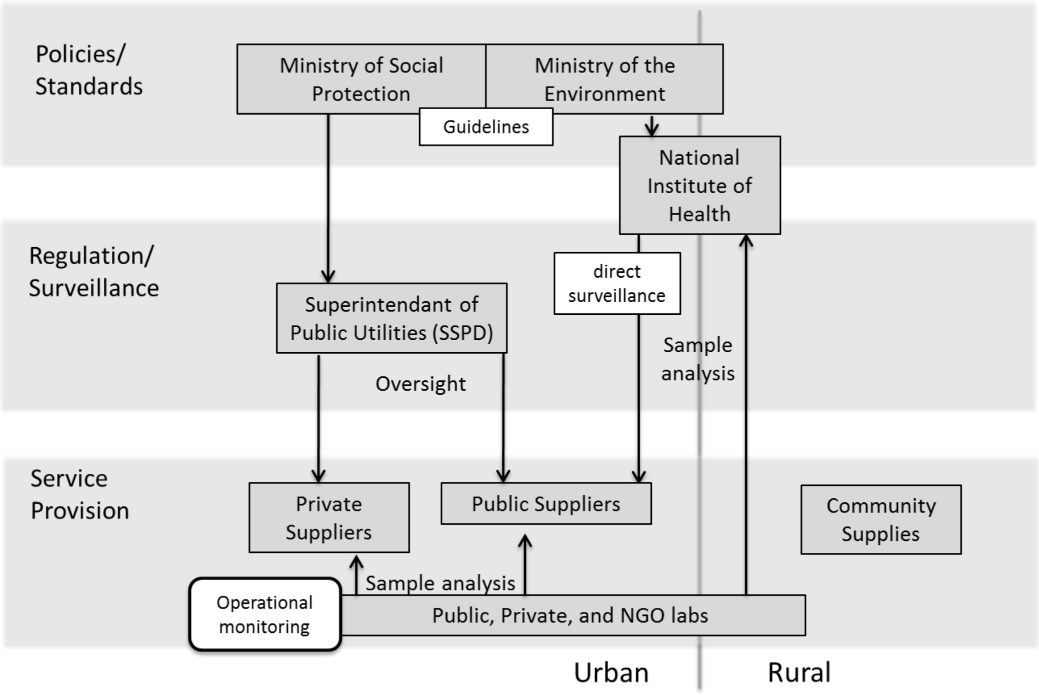

2.2. Scenarios

The large supply operational monitoring scenario in Colombia includes EPS-managed piped supplies serving over 100,000. The small supply operational monitoring scenario includes small EPS-managed supplies serving fewer than 100,000 people. Surveillance monitoring includes monitoring by the INS, covering all water supplies in Colombia. Quantitative values for monitoring scenarios in Colombia are presented in Table S2.

Table S2. Colombia scenarios parameters.

Monitoring Type	Parameter	Large Supply Operational	Small Supply Operational	Surveillance
	target pop	13,836,161	7,244,193	45,000,000
prescribed	tests/1000 capita	3.25	8.98	1.71
	cost/1000 capita	$24.44	$82.10	$15.63
extrapolated	tests/1000 capita	5.42	5.38	0.97
	cost/1000 capita	$40.74	$49.17	$8.86

3. India—National Level

Sector Description

In India, the water sector is primarily managed at the state level rather than the national level. The role of the national government of India is in setting guidelines and providing funding to the states. The Bureau of Indian Standards (BIS) sets standards or drinking water quality monitoring, including

how sampling should be conducted, how testing should be conducted and which tests should be used, and requirements for final drinking water quality.

The Ministry of Urban Development (MoUD) is one of two ministries in the national government responsible for creating recommendations and frameworks for water supply and monitoring at the state level, and for partial funding of state governments' drinking water plans. The MoUD is specifically concerned with urban water supplies. The MoUD does not create legally binding frameworks, but is influential due to its role as the primary funder of urban water projects.

The Ministry of Rural Development (MoRD) is parallel to the MoRD. The MoRD is specifically concerned with rural water supply and monitoring. The MoRD does not have legal jurisdiction over practices in the individual states, but they are the most influential organization in relation to rural water supply and monitoring due to their role as the primary funder of state level projects. Roles and responsibilities in the water supply sector in India at the national level are displayed in Figure S3.

Figure S3. India institutional map.

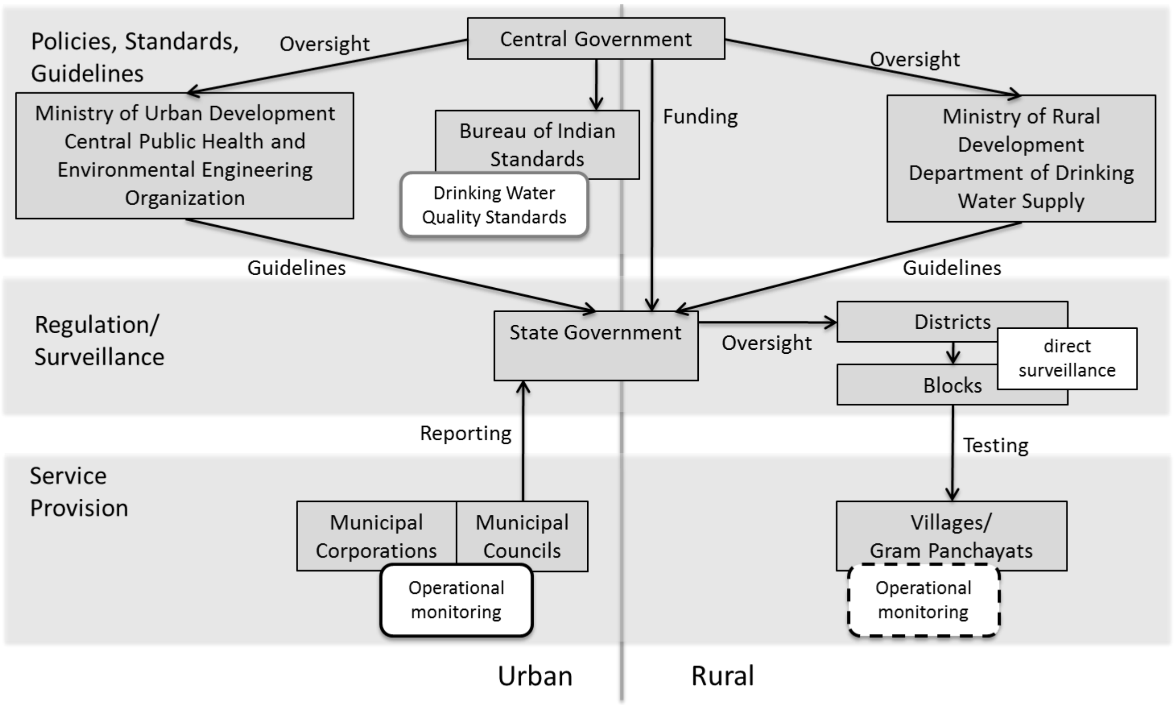

4. India—Maharasthra

4.1. Sector Description

The Public Health Department (PHD) is involved in policy modification; the final decisions regarding policy modifications must be approved by the Directorate of Health Services in the PHD. The PHD is also in charge of the network of public health laboratories, including the state, district public health laboratories and the rural hospital laboratories, which perform the majority of water quality testing in Maharashtra. The state department of urban development oversees the 23 municipal corporations that provide and monitor drinking water for the largest cities in Maharastra. There are 226 smaller cities and towns which have water supply provided and monitored by their municipal

councils under the oversight of the PHD. Rural supply is monitored by Multi-Purpose Workers, overseen by the PHD. Roles and responsibilities in the water supply sector in Maharashtra are displayed in Figure S4.

Figure S4. Maharashtra institutional map.

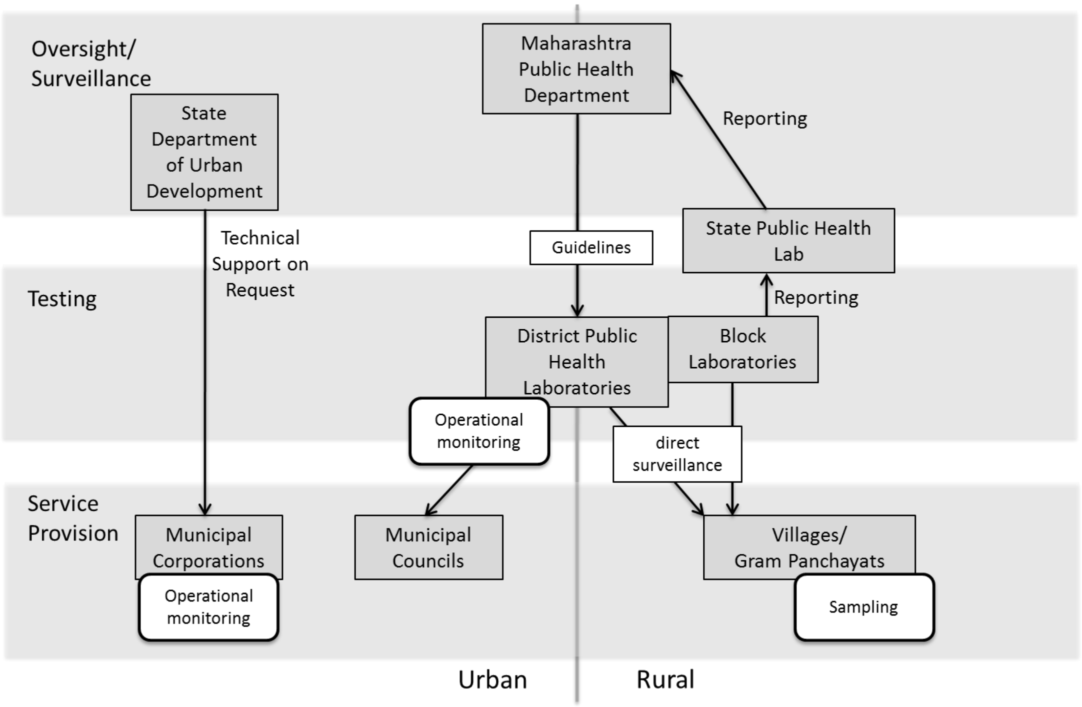

4.2. Scenarios

The large supply operational monitoring scenario in Maharasthra includes the six largest Municipal Corporations (Mumbai, New Mumbai, Pimpri, Pune, Solapur, and Thane), which perform their own water quality monitoring and operate their own water quality laboratories. The small supply operational monitoring scenario includes 17 smaller municipal corporations, and 226 municipal councils. The 17 smaller municipal corporations and 226 municipal councils use DPHLs for analysis of samples from their water supplies. Surveillance covers rural water supply, and is carried out by the PHD, through their state laboratory, block laboratories, and public health laboratories. Quantitative values for monitoring scenarios in Maharashtra are presented in Table S3.

Table S3. Maharashtra scenarios parameters.

Monitoring Type	Parameter	Large Supply Operational	Small Supply Operational	Surveillance
	target pop	18,368,426	19,493,831	55,777,647
prescribed	tests/1000 capita	1.38	1.78	21.51
	cost/1000 capita	$7.48	$10.30	$124.65
extrapolated	tests/1000 capita	1.38	1.78	21.51
	cost/1000 capita	$7.48	$10.30	$124.65

5. India—Uttar Pradesh

5.1. Sector Description

The Uttar Pradesh Jal Nigam (UPJN) is responsible for oversight of drinking water and sanitation in Uttar Pradesh. The UPJN follows the guidelines and manuals created at the national level by the MoRD and the MoUD. The UPJN is responsible for building water-related infrastructure, but transfers water projects to the local Jal Sansthan or Panchayati Raj Institution for operation and maintenance. Jal Sansthans (JS) act as water boards in five of the largest cities in Uttar Pradesh each have their own Jal Sansthan. The Jal Sansthans are responsible for treatment, distribution, and monitoring of drinking water in these cities. The State Health Institute (SHI) is primary of the two laboratories that tests drinking water samples from the Jal Sansthans in the state. The Indian Institute of Toxicology Research processes some samples for the UPJN for surveillance purposes. Water Works (WWs) exist in 18 small cities and towns, where they manage, operate, and maintain drinking water supply for these towns. None of the 18 WWs test for microbiological indicators, but they occasionally send water samples to UPJN laboratories for microbiological testing. The State Water and Sanitation Mission supports the UPJN in their role in policy development and regulation of water suppliers.

The gram panchayats (GPs) are the smallest unit of local government within a state and are composed of varying numbers of villages. Wide-scale use of field testing kits is being piloted in Uttar Pradesh, and will rely heavily on community participation through the gram panchayats. Village Water and Sanitation Committees (VWSCs) are five person teams made up of individuals from a gram panchayat who are responsible for testing rural water supplies using H_2S field kits. Some VWSCs are already operating, and additional VWSC workers are being recruited and trained to expand rural testing. Roles and responsibilities in the water supply sector in Uttar Pradesh are displayed in Figure S5.

Figure S5. Uttar Pradesh institutional map.

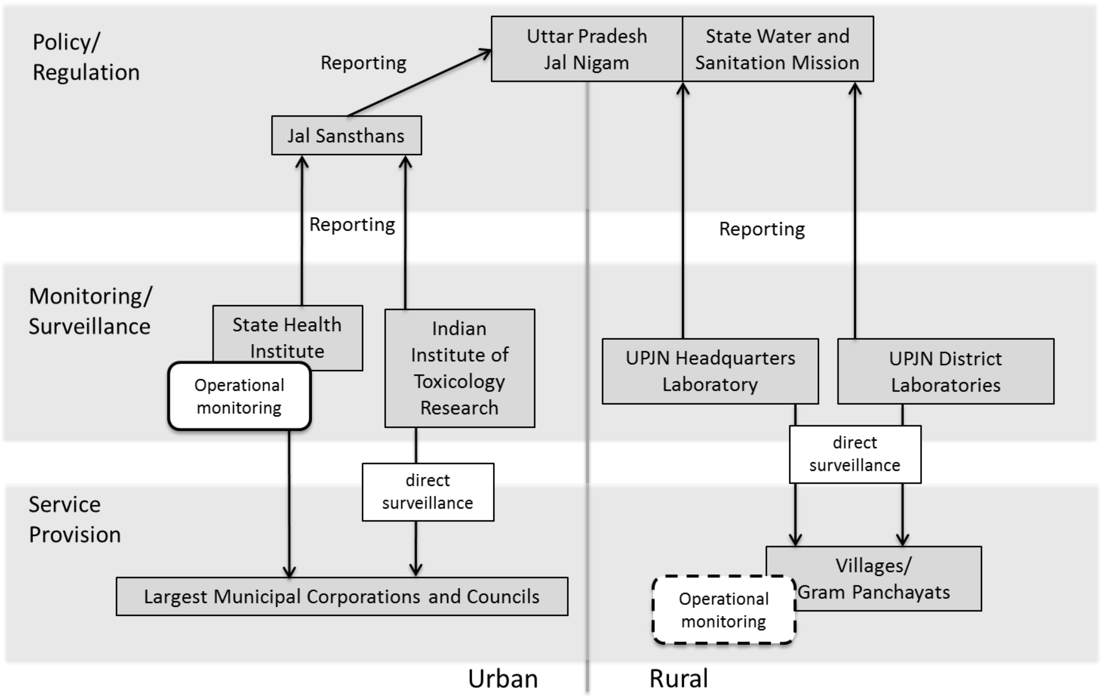

5.2. Scenarios

The large supply operational monitoring scenario in Uttar Pradesh included the largest five cities: Allahabad, Agra, Kanpur, Lucknow, and Varansi. Analysis of samples from these cities is carried out by the State Health Institute. The small supply operational monitoring scenario includes approximately 630 smaller cities and towns, and 2 million rural supplies. There was no evidence of monitoring for the 630 cities and towns, while some of the rural supplies were monitored by village committees using field tests. Surveillance is carried out by the UPJN and Indian Institute of Toxicology Research for urban areas, and by the UPJN using their district laboratories for rural areas. Quantitative values for monitoring scenarios in Uttar Pradesh are presented in Table S4.

Table S4. Uttar Pradesh scenarios parameters.

Monitoring Type	Parameter	Large Supply Operational	Small Supply Operational	Surveillance
	target pop	8,538,593	157,186,224	131,283,554
prescribed	tests/1000 capita	1.20	28.36	1.68
	cost/1000 capita	$7.65	$31.15	$8.03
extrapolated	tests/1000 capita	0.06	0.08	0.84
	cost/1000 capita	$0.41	$0.08	$4.02

6. India—West Bengal

6.1. Sector Description

Urban water supply in West Bengal is operated by the municipalities, and overseen by Urban Local Bodies. Municipal corporations in the four largest cities operate their own laboratories for monitoring drinking water quality. One such example is the Kolkata Municipal Corporation (KMC) which is the water supplier for the city of Kolkata. The KMC is responsible for treating, supplying and monitoring the drinking water for Kolkata. The 236 smaller municipalities provide water but do not monitor it themselves. The Kolkata Municipal Development Agency (KMDA) provides laboratory facilities for monitoring of these supplies.

The Public Health Engineering Department (PHED) is responsible for providing drinking water to rural areas, overseeing the functioning of the block level water testing laboratories, maintaining the PHED water quality online database, and analyzing the data in the PHED database. The Department of Panchayats and Rural Development is responsible for training the water sample collectors (Gram Panchayat facilitators), organizing payments from the state to the Zilla Parishad and maintaining the online water quality database and analyzing the data. Zilla Parishads are the district level local government body responsible for moving funds from the state level organizations to the block level laboratories, and for paying the GP facilitators who collect water samples. Block laboratories (BLs) are managed in two ways in West Bengal: those managed by NGOs (80) and those managed by the PHED (34). There is very little difference between the two laboratory types in practice, though they have different management and PHED laboratories are in general newer than NGO laboratories. All of the samples collected by the gram panchayat facilitators are tested at the block laboratories. There is

generally one block laboratory for every three blocks. Roles and responsibilities in the water supply sector in West Bengal are displayed in Figure S6.

Figure S6. West Bengal institutional map.

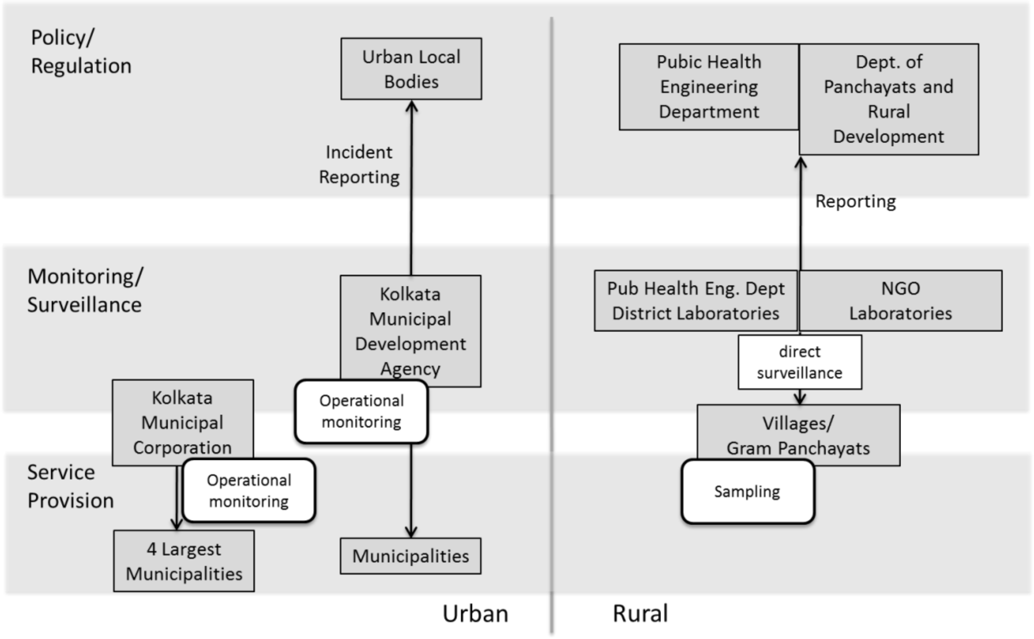

6.2. Scenarios

The large supply operational monitoring scenario includes monitoring water provided and monitored by the municipal corporations in the four largest cities: Kolkata, Howrah, Silguri, and Assansol. The small supply operational monitoring scenario includes monitoring in 236 smaller municipalities. There are 4 labs that monitor the drinking water for small cities and towns in West Bengal: the KMDA central lab, the All India Institute of Hygiene and Public Health (AIIHPH) lab, the State Pollution Control Board (SPCB) lab, and the Jadavpur University lab. The surveillance scenario includes monitoring of rural drinking water quality overseen by the PHED. Water Samples are collected from each water source 1 to 2 times per year by gram panchayat facilitators, who deliver them to one of the 80 NGO run block labs and 35 PHED run block labs for testing. Quantitative values for monitoring scenarios in West Bengal are presented in Table S5.

Table S5. West Bengal scenarios parameters.

Monitoring Type	Parameter	Large Supply Operational	Small Supply Operational	Surveillance
	target pop	14,745,440	7,681,811	57,748,818
prescribed	tests/1000 capita	1.20	2.50	16.29
	cost/1000 capita	$6.52	$21.76	$94.37
extrapolated	tests/1000 capita	0.75	0.12	1.80
	cost/1000 capita	$4.10	$1.07	$10.42

7. Jordan

7.1. Sector Description

The Ministry of Health (MOH) is responsible for participating in formation of water quality standards, monitoring standards and regulation, as well as responsible for surveillance monitoring of all water supply in Jordan. The Water Authority of Jordan (WAJ) is the central actor in the water sector in Jordan. They are a participating organization in the formation of regulations and standards. They act as supplier for all of Jordan excepting Amman and Aqaba. They are responsible for monitoring all drinking water in Jordan excepting the Aqaba governorate. In Amman they are responsible for 20% of the total yearly samples taken from the distribution system. They also act as regulator for water supply in Jordan, receiving water quality reports from the Aqaba Water Company, Miyahuna, and the Ministry of Health. Miyahuna is a private water supplier for the city of Amman. The Aqaba Water Company supplies water for the Aqaba governorate, and contracts Ben Hayyan laboratories for sample analysis. The Jordan Institution for Standards and Metrology (JISM) is the standards board for all of Jordan, and their activities in the water sector extend beyond standards. In addition to setting water quality standards and monitoring standards, they set procedures. They also organize and chair the Permanent Technical Committee, which meets every 5 years to create and amend any water policy, regulation, or standards. The most relevant document published by the JISM is JS286:2008, the national standard for drinking water quality, which includes sampling procedures and testing procedures. Roles and responsibilities in the water supply sector in Jordan are displayed in Figure S7.

Figure S7. Jordan institutional map.

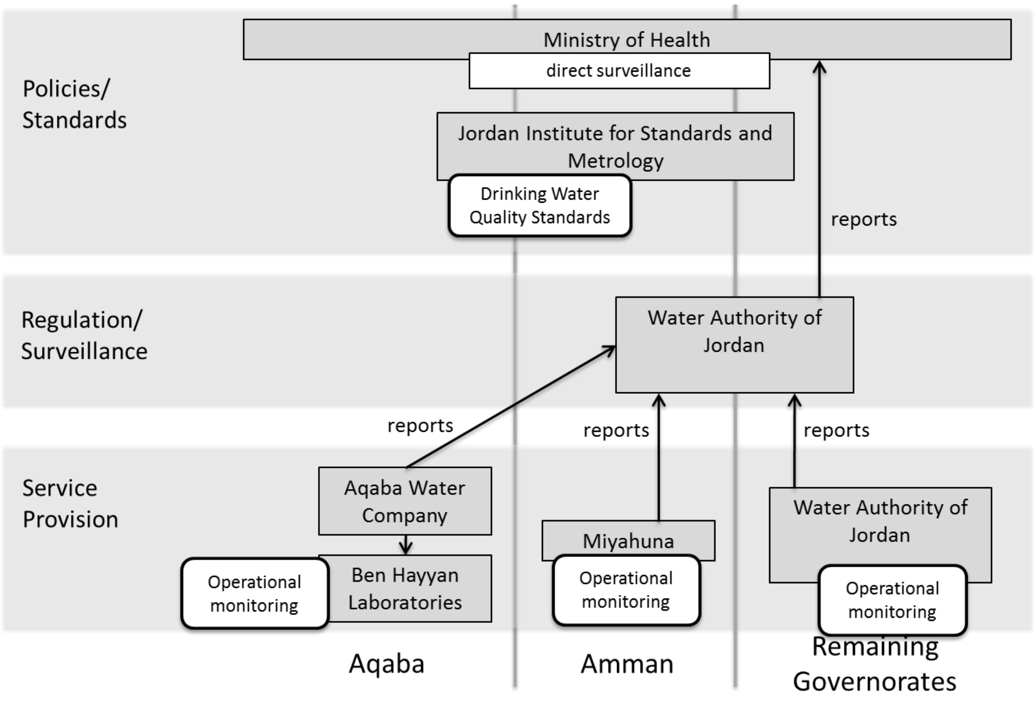

7.2. Scenarios

The large supply operational monitoring scenario in Jordan includes monitoring in Amman by Miyahuna and WAJ. The small supply operational monitoring scenario includes monitoring by WAJ of all other water supply in the country, which is 80% urban, and monitoring of the Aqaba water supply by Ben Hayyan labs. The surveillance scenario includes monitoring by the MOH of the all water supply except in the Aqaba governorate. Quantitative values for monitoring scenarios in Jordan are presented in Table S6.

Table S6. Jordan scenarios parameters.

Monitoring Type	Parameter	Large Supply Operational	Small Supply Operational	Surveillance
	target pop	2,800,000	6,514,000	6,407,000
prescribed	tests/1000 capita	2.28	2.31	2.64
	cost/1000 capita	$15.06	$19.01	$21.75
extrapolated	tests/1000 capita	2.16	3.92	2.64
	cost/1000 capita	$14.26	$32.25	$21.75

8. Peru

8.1. Sector Description

The general directorate of environmental health (DIGESA—Direccion General de Salud Ambiental) is the authority within the ministry of health responsible for the development of drinking water policy and standards, supervision and management of drinking water provision, and establishing standards for drinking water quality monitoring. The regional branches of DIGESA (DIRESA—Direcciones Regionales de Salud) are responsible for supervising drinking water surveillance. The National Superintendent of Sanitation Services (SUNASS—Superintendencia Nacional de Servicios de Saneamiento) is a regulatory body tasked with oversight of water service providers. There are 45 water service providers (EPS—Empresa Prestadora de Servicios) that provide water to their designated locals throughout Peru. Roles and responsibilities in the water supply sector in Peru are displayed in Figure S8.

Figure S8. Peru institutional map.

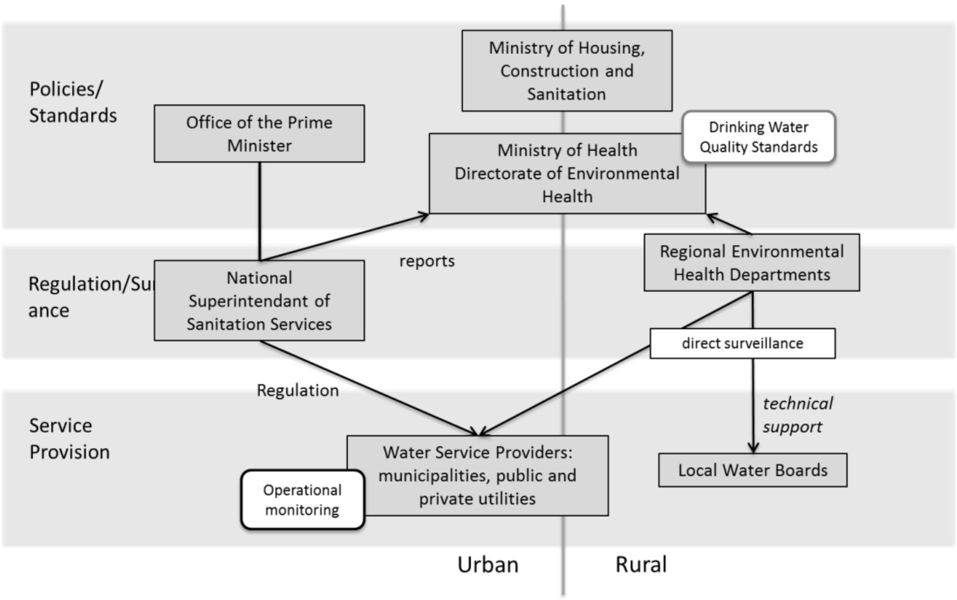

8.2. Scenarios

The large supply operational monitoring scenario in Peru includes water service providers with over 40,000 connections. The small supply operational monitoring scenario includes suppliers with less than 40,000 connections. The surveillance scenario includes monitoring by the Ministry of Health of supplies that serve less than 5000 people. Quantitative values for monitoring scenarios in Peru are presented in Table S7.

Table S7. Peru scenarios parameters.

Monitoring Type	Parameter	Large Supply Operational	Small Supply Operational	Surveillance
	target pop	14,201,321	3,120,810	9,340,159
prescribed	tests/1000 capita	2.69	2.48	33.41
	cost/1000 capita	$17.34	$19.99	$269.66
extrapolated	tests/1000 capita	missing data		0.68
	cost/1000 capita			$5.50

9. South Africa

9.1. Sector Description

The Department of Water Affairs (DWA) is in charge of oversight, support, monitoring, and capacity building of Water Services Authorities (WSA). This role includes contributing to standards development and performing audit-based surveillance. The South African Bureau of Standards (SABS) has the role of defining standards for drinking water quality and monitoring. The Environmental Health departments within the Department of Health play a role in monitoring, by providing environmental health practitioners to carry out sample collection. Emanti is a private firm, hired by the national government. They are a company that provides the platform for municipalities to capture water quality

data, analyze data, interpret data, and generate or view reports. They are heavily involved on the Local Municipal level. Water Service Authorities are the main organizations responsible for water provision. A WSA can exist at the metropolitan (6), district (48), or local (231) municipality level. Water Service Providers (WSP) can be both public and private, and are contracted by WSAs for some water supplies to take on the responsibility of water provision. Roles and responsibilities in the water supply sector in South Africa are displayed in Figure S9.

Figure S9. South Africa institutional map.

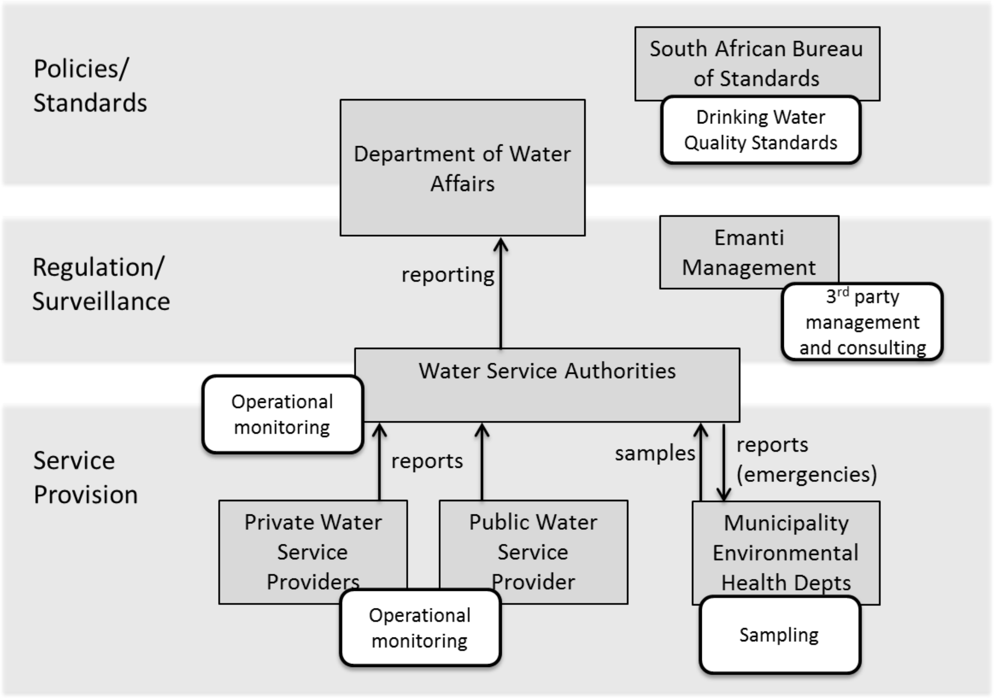

9.2. Scenarios

The large supply operational monitoring scenario in South Africa includes the 34 largest urban centers in, with an average population of 700,000 people per water supply, which are monitored by a WSA, and a WSP in addition in some cases. The small supply operational monitoring scenario is made up mostly point-source supplies, with a few smaller piped supplies, which are predominantly monitored by WSA, but in a few cases a WSP may exist as well. Traditional surveillance monitoring doesn't occur in South Africa, though the government is involved in operational monitoring of all water supply in the country. Quantitative values for monitoring scenarios in South Africa are presented in Table S8.

Table S8. South Africa scenarios parameters.

Monitoring Type	Parameter	Large Supply Operational	Small Supply Operational	Surveillance
	target pop	24,228,853	25,730,998	51,200,000
prescribed	tests/1000 capita	2.10	1.40	0
	cost/1000 capita	$20.66	$14.13	$0.00
extrapolated	tests/1000 capita	2.09	1.30	0
	cost/1000 capita	$20.54	$13.02	$0.00

10. Uganda

10.1. Sector Description

The Ministry of Water and Environment (MWE) has the responsibility for setting national policies and standards for drinking water, managing and regulating water resources, and determining priorities for water development and management. The Directorate of Water Resources Management is has the responsibility for developing and maintaining national laws, policies and regulations; and managing, monitoring, and regulation of water resources. The Department of Water Quality Management is charged with managing the quality of the country's water resources and various uses. It is also responsible for compliance monitoring of urban and rural water systems. They are charged with conducting surveillance and compliance monitoring though the frequency at which surveillance monitoring is conducted is unknown. The National Bureau of Standards is responsible for setting standards for drinking water quality monitoring.

The National Water and Sewerage Corporation (NWSC) provides and monitors water and sewerage services to 23 large urban towns. Water Authorities (WA) of small towns (towns with populations above 5000 and outside the jurisdiction of NWSC) are mandated by the MWE to provide and monitor piped water supply services within their localities. The Association of Private Water Operators are 20 private operators contracted to manage WA schemes. District Water Offices supply water to rural areas mainly by use of point water sources. Roles and responsibilities in the water supply sector in Uganda are displayed in Figure S10.

Figure S10. Uganda institutional map.

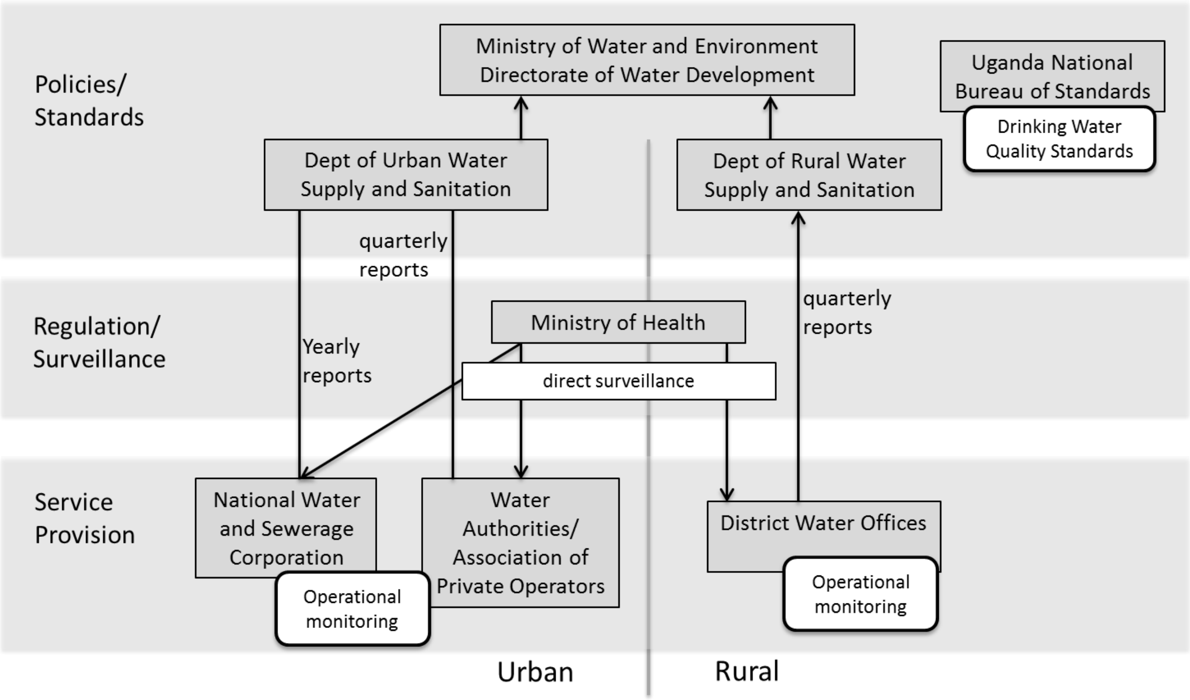

10.2. Scenarios

The large supply operational monitoring scenario in Uganda includes the 23 largest towns in the country, which are supplied and monitored by the NWSC. The small supply operational monitoring scenario includes water supplied by the water authorities, which thus does not include any settlements with less than 5000 people. The surveillance scenario includes monitoring by the ministry of health for the entire population. Quantitative values for monitoring scenarios in Uganda are presented in Table S9.

Table S9. Uganda scenarios parameters.

Monitoring Type	Parameter	Large Supply Operational	Small Supply Operational	Surveillance
	target pop	2,196,393	1,136,325	32,709,865
prescribed	tests/1000 capita	6.34	26.14	7.40
	cost/1000 capita	$39.27	$204.47	$57.91
extrapolated	tests/1000 capita	missing data		
	cost/1000 capita			

Supplement 2—Data Sources and Assumptions

Scenario	Prescribed number of tests	Extrapolated number of tests	Material costs
		**CAMBODIA**	
Large supply operational	*Cambodian National Drinking Water Quality Standards*, Ministry of Industry, Mines, and Development, 2004.	One interview account; one laboratory sampling and testing report.	$2.25 [10]
Small supply operational		One interview account; multiple laboratory sampling and testing reports.	$1.04 [10]
Surveillance	NA	NA	NA
		**COLOMBIA**	
Large supply operational	*Resolution 2115*, Ministry of Social Protection, 2007; water supplier data, Superintendent of Public Utilities, 2010.	Online database, Superintendent of Public Utilities, 2010.	$2.25 [10]
Small supply operational			
Surveillance		Online database, National Institute of Health, 2010.	
		**INDIA—MAHARASHTRA**	
Large supply operational	*Manual for the Supply and Treatment of Drinking Water*, Central Public Health and Environmental Engineering Organization (CPHEEO), 2007; national census, Ministry of Home Affairs, 2001.	Online database of compliance figures; *Manual for the Supply and Treatment of Drinking Water*, CPHEEO, 2007; national census, Ministry of Home Affairs, 2001.	$1.62 [9]
Small supply operational			
Surveillance	One interview account (4 tests annually for each of ~300,000 non-piped supplies is the goal).	Two interview accounts (each source is tested 4 times annually; and each multi-purpose worker collects ~10 samples per year). These two accounts produce estimates of 1,200,000 and 1,416,360 tests annually. The lower number is used in analysis.	
		**INDIA—UTTAR PRADESH**	
Large supply operational	*Manual for the Supply and Treatment of Drinking Water*, CPHEEO, 2007; national census, Ministry of Home Affairs, 2001.	1-week sampling and testing report.	$1.62 [9]
Small supply operational	*Manual for the Supply and Treatment of Drinking Water*, CPHEEO, 2007; national census, Ministry of Home Affairs, 2001; one interview account (2 tests annually for each of ~2,200,000 is the goal for non-piped supply).	Multiple interview accounts (that ~10% of the National Rural Drinking Water Program's data on the Integrated Management Information System website comes from this scenario.	$1.62 for piped, $0.62 for non-piped [9]
Surveillance	One interview account (annual testing of 10% of all water supplies is the surveillance goal).	One interview account (~100,000-120,000 samples are analyzed per year, which is ~5% of water supplies).	$0.62 [9]
		**INDIA—WEST BENGAL**	
Large supply operational	*Manual for the Supply and Treatment of Drinking Water*, CPHEEO, 2007; national census, Ministry of Home Affairs, 2001.	One interview account (compliance for Kolkata).	$1.62 [9]
Small supply operational		One interview account (4 tests annually for each town); national census, Ministry of Home Affairs, 2001.	
Surveillance	One interview account (2 tests annually per water supply is the goal).	6-month laboratory sampling and testing report.	

**Scenario**	**Prescribed number of tests**	**Extrapolated number of tests**	**Material costs**
		**JORDAN**	
Large supply operational	Annual sampling and testing plan, Miyahuna, 2010; *Standard 286*, Jordan Institute of Standards and Metrology (JISM), 2001; *Population and Housing Census*, Department of Statistics, 2004.	Laboratory sampling and testing reports, Miyahuna, 2008-2010.	$1.62 [9]
Small supply operational	*Standard 286*, JISM, 2001;	One interview account.	
Surveillance	*Population and Housing Census*, Department of Statistics, 2004.	One interview account.	
		**PERU**	
Large supply operational	Internal guidelines, National Superintendent of Sanitation Services, 2010.	NA	$1.62 [9]
Small supply operational			
Surveillance	*Regulation of the official physical, chemical, and bacteriological requirements that drinking water should meet to be considered potable,* government of Peru, 1946; national census, National Institute of Statistics and Information, 2007.	Three interview accounts.	
		**SOUTH AFRICA**	
Large supply operational	*South Africa National Standard 241*, South Africa Bureau of Standards, 2011;	Multiple interview accounts; Blue Drop online databases, Department of Water Affairs, 2010.	$2.25 [10]
Small supply operational	*South Africa National Census*, Statistics South Africa, 2001.	Three interview accounts.	$2.25 [10] and $1.62 [9]
Surveillance	NA	NA	NA
		**UGANDA**	
Large supply operational	Internal guidelines, National Water and Sewerage Corporation, 2010.	NA	$2.25 [10]
Small supply operational	Multiple laboratory sampling and testing reports.	NA	
Surveillance	*Uganda Standard 201 - Drinking (potable) water specification*, Uganda National Bureau of Standards, 2008; *Water and Environment Sector Performance Report*, Ministry of Water & Energy, 2009.	NA	

**Scenario**	**Labor ($/test)**	**Assumptions and methods used**	**Transport ($/test)**	**Assumptions and methods used**
**CAMBODIA**				
Large supply operational	$4.00	Based on multiple interviews and observations.	$4.14	Based on interview account of compensation to samplers.
Small supply operational	$4.00	Based on multiple interviews and observations.	$4.14	Value taken from Cambodia large supply operational.
Surveillance	NA	NA	NA	NA
**COLOMBIA**				
Large supply operational	$3.51	Assumes sampling collection and testing rates from Jordan, with salary adjusted to similar job offerings in Colombia.	$1.75	Value taken from Jordan large supply operational.
Small supply operational	$3.51	Value taken from Colombia large supply operational.	$3.38	Value taken from Jordan small supply operational.
Surveillance	$3.51	Value taken from Colombia large supply operational.	$3.38	Value taken from Jordan small supply operational.
**INDIA—MAHARASHTRA**				
Large supply operational	$2.06	Value taken from West Bengal large supply operational.	$1.75	Value taken from Jordan large supply operational.
Small supply operational	$1.50	Value taken from West Bengal surveillance.	$2.67	Value taken from West Bengal small supply operational.
Surveillance	$1.50	Value taken from West Bengal surveillance.	$2.67	Value taken from West Bengal surveillance. This estimate may be high for Maharashtra, as there are more labs available.
**INDIA—UTTAR PRADESH**				
Large supply operational	$2.06	Value taken from West Bengal large supply operational.	$2.67	Value taken from West Bengal small supply operational. In Uttar Pradesh, one lab serves the 5 largest cities, so sample collection functions similarly to small supplies.
Small supply operational	$0.15; $1.5	Non-piped: assumes that field-based testing requires 10% time for training volunteers and delivering test devices; piped: see assumptions from West Bengal.	$0.267; $2.67	Non-piped: assumes that field-based testing requires 10% transportation for training volunteers and delivering test devices; piped: see assumptions from West Bengal.
Surveillance	$1.50	Value taken from West Bengal surveillance.	$2.67	Value taken from West Bengal surveillance.
**INDIA—WEST BENGAL**				
Large supply operational	$2.06	Based on interviews and laboratory observations from Kolkatta, assumes that testing involves lab chemists, department analysts, and assistant analysts at three different salaries quoted from laboratory guidelines. Chemists are assumed to complete 2500 tests in a year, and analysts are assumed to spend half their time on microbiological monitoring.	$1.75	Value taken from Jordan large supply operational.
Small supply operational	$1.50	Value taken from West Bengal surveillance.	$2.67	Based on UNICEF guidelines for West Bengal.
Surveillance	$1.50	Based on UNICEF guidelines for West Bengal, and multiple interviews. Assumes samplers collect 5 samples in a day at a $500 salary, and lab staff get paid $1.1 per sample analyzed.	$2.67	Based on UNICEF guidelines for West Bengal.

**Scenario**	**Labor ($/test)**	**Assumptions and methods used**	**Transport ($/test)**	**Assumptions and methods used**
		**JORDAN**		
Large supply operational	$3.23	Value taken from Jordan small supply operational.	$1.75	Based on an interview and observation of a sampling trip, assumes 5 miles travelled per sample; and, based on American Automobile Association formulas, assumes $0.35 per mile.
Small supply operational	$3.23	Based on interviews, laboratory observations, and salaries for job offerings in similar government jobs, assumes 6052 samples collected per sampler per year at a salary of $5948.57 per year, and 2650 samples tested per lab technician per year at a salary of $8194.29 per year.	$3.38	Based on an interview and observation of a sampling trip, assumes 11.8 miles travelled per sample; and, based on American Automobile Association formulas, assumes $0.35 per mile.
Surveillance	$3.23	Value taken from Jordan small supply operational.	$3.38	Value taken from Jordan small supply operational.
		**PERU**		
Large supply operational	$3.07	Assumes sampling collection and testing rates from Jordan, with salary adjusted to similar job offerings in Peru.	$1.75	Value taken from Jordan large supply operational.
Small supply operational	$3.07	Value taken from Peru large supply operational.	$3.38	Value taken from Jordan small supply operational.
Surveillance	$3.07	Value taken from Peru large supply operational.	$3.38	Value taken from Jordan small supply operational.
		**SOUTH AFRICA**		
Large supply operational	$5.82	Assumes sampling collection and testing rates from Jordan, with salary adjusted to similar job offerings in South Africa.	$1.75	Value taken from Jordan large supply operational.
Small supply operational	$5.82	Value taken from South Africa large supply operational.	$3.38	Value taken from Jordan small supply operational.
		**UGANDA**		
Large supply operational	$2.20	Assumes sampling collection and testing rates from Jordan, with salary adjusted to similar job offerings in Uganda.	$1.75	Value taken from Jordan large supply operational.
Small supply operational	$2.20	Value taken from Uganda large supply operational.	$3.38	Value taken from Jordan small supply operational.
Surveillance	$2.20	Value taken from Uganda large supply operational.	$3.38	Value taken from Jordan small supply operational.

© 2014 by the authors; licensee MDPI, Basel, Switzerland. This article is an open access article distributed under the terms and conditions of the Creative Commons Attribution license (http://creativecommons.org/licenses/by/3.0/).
